# The Role of Signal Transducer and Activator of Transcription 3 (STAT3) and Its Targeted Inhibition in Hematological Malignancies

**DOI:** 10.3390/cancers10090327

**Published:** 2018-09-13

**Authors:** Loukik Arora, Alan Prem Kumar, Frank Arfuso, Wee Joo Chng, Gautam Sethi

**Affiliations:** 1 Department of Pharmacology, Yong Loo Lin School of Medicine, National University of Singapore, Singapore 117600, Singapore; arora.loukik@u.nus.edu (L.A.); csiapk@nus.edu.sg (A.P.K.); 2 Cancer Science Institute of Singapore, Centre for Translational Medicine, 14 Medical Drive, #11-01M, Singapore 117599, Singapore; mdccwj@nus.edu.sg; 3 Medical Science Cluster, Yong Loo Lin School of Medicine, National University of Singapore, Singapore 117600, Singapore; 4 Curtin Medical School, Faculty of Health Sciences, Curtin University, Perth, WA 6102, Australia; 5 National University Cancer Institute, National University Health System, Singapore 119074, Singapore; 6 Stem Cell and Cancer Biology Laboratory, School of Biomedical Sciences, Curtin Health Innovation Research Institute, Curtin University, Perth, WA 6102, Australia; frank.arfuso@curtin.edu.au; 7 Department of Hematology-Oncology, National University Cancer Institute, National University Health System, Singapore 119074, Singapore; 8 School of Pharmacy and Biomedical Sciences, Curtin Health Innovation Research Institute, Curtin University, Perth, WA 6009, Australia

**Keywords:** STAT3, hematological malignancies, targeted inhibition, proliferation, anti-apoptosis

## Abstract

Signal transducer and activator of transcription 3 (STAT3), a member of the STAT protein family, can be phosphorylated by receptor-associated Janus kinases (JAKs) in response to stimulation by cytokines and growth factors. It forms homo- or heterodimers that can translocate to the cell nucleus where they act as transcription activators. Constitutive activation of STAT3 has been found to be associated with initiation and progression of various cancers. It can exert proliferative as well as anti-apoptotic effects. This review focuses on the role of STAT3 in pathogenesis i.e., proliferation, differentiation, migration, and apoptosis of hematological malignancies viz. leukemia, lymphoma and myeloma, and briefly highlights the potential therapeutic approaches developed against STAT3 activation pathway.

## 1. Introduction

Signal Transducers and Activators of Transcription (STATs), were first discovered in 1994 and found to be involved in interferon (IFN)-triggered transcription regulation [1,2,3]. To date, seven different STAT proteins, namely STAT1, -2, -3, -4, -5a, -5b, and -6, have been discovered in mammalian cells [2,3,4,5]. The STAT family proteins transduce intracellular and extracellular signals that mediate multiple cellular functions including those related to survival, growth and proliferation, and angiogenesis [6,7]. STAT family members are generally localized in the cytoplasmic compartment of the cell, in an inactive state, either as monomers or as latent (unphosphorylated) dimers [8] that can be activated by various stimuli such as cytokines and growth factors [9,10,11]. STATs are activated by phosphorylation on tyrosine by Janus kinases (JAKs), Src family members, as well as growth factor receptors such as epidermal growth factor receptor (EGFR) and platelet-derived growth factor receptor (PDGFR) [1,10,12]. The dimer thereafter enters the nucleus via interaction with importins and can bind target genes [7].

## 2. Domain Structure and Signaling Cascade of STAT3

STAT3 is an important member of STAT family that regulates gene transcription by relaying signals from activated plasma membrane receptors to the nucleus in response to various stimuli [13,14].

### 2.1. Structure of STAT3

**Figure 1:**
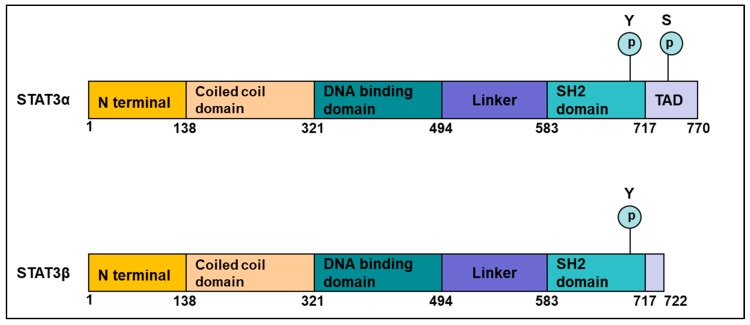
The molecular structure of the two STAT3 isoforms. The STAT3 molecule consists of several distinct domains:(i) the N-terminal domain containing an oligomerization and a coiled-coil domain, (ii) the DNA-binding domain, (iii) the linker domain, (iv) the Src homology 2 (SH2) domain, and (v) the C-terminal Transactivation domain (TAD). STAT3α is the full-length STAT3 protein, with a TAD at the C terminal that has a serine residue at position 727. STAT3β is the truncated STAT3 protein, without the TAD.

The structure of the STAT3 molecule has several distinct functional domains (Figure 1):

- The N-terminal domain—composed of an oligomerization and a coiled-coil domain.
- The DNA-binding domain—that can recognize a specific molecular motif in the DNA.
- A linker domain.
- The Src homology 2 (SH2) domain—important for the formation of dimers; phospho-tyrosine 705 is important for this dimerization.
- The C-terminal transactivation domain—This domain differs between the α and β isoforms, with the β form having a unique truncated C-terminal sequence. STAT3 protein also has a Ser 727 phosphorylation site, which though less studied than Tyr 705, is important for regulating STAT3 activation by serine/threonine kinases such as the MAPKs (mitogen-activated protein kinases) [15].

### 2.2. STAT3 Signaling Cascade

**Figure 2:**
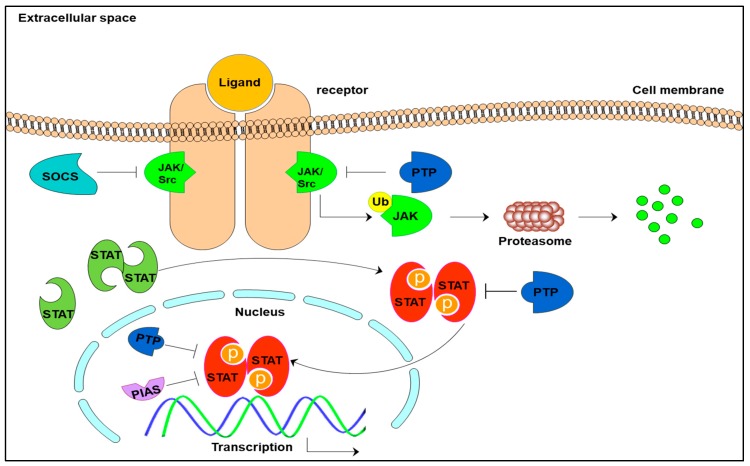
A schematic representation of STAT3 signaling cascade. STAT proteins are present in an inactive (unphosphorylated latent dimers) form in the cytoplasm. Upon ligand binding, the receptors dimerize to recruit upstream JAK/Src kinases. Phosphorylated tyrosine residues on the receptors provide a docking site to STATs and bring about their phosphorylation and activation. Phosphorylated STATs upon translocation to nucleus bind onto the consensus sequence and thereby regulate gene transcription.

STAT3, activated by phosphorylation, is a downstream target of several plasma membrane surface receptors that can be activated by a plethora of soluble mediators, including interleukins (IL-2, I-L3, IL-5, Il-6-IL-7, IL-9-IL11), other cytokines (G-CSF, EGF, PDGF), and several hormones (growth hormone, prolactin, leptin) [16,17]. The activation of protein is brought about by phosphorylation of tyrosine residue at 705 by either receptor tyrosine kinases (RTK) such as EGFR, PDGFR, fibroblast growth factor receptor (FGFR), insulin-like growth factor receptor (IGFR) or receptor associated kinases such as JAK, and non-receptor tyrosine kinases like Src and Abl [18,19]. Ligation of these cell surface receptors causes stimulation of either: (1) intrinsic tyrosine kinase activity or, (2) receptor chain aggregation and thereby kinase activation (Figure 2). Although STAT3 dimerization is usually depicted after phosphorylation, increasing evidences suggest the possibility of an existence of preformed latent STAT3 dimers. The Y705 phosphorylation induces the activation of these latent STAT3 dimers [8,20]. The dimers, both latent and activated can shuttle between nucleus and cytoplasm. Latent or unphosphorylated dimers shuttle via nucleoporins Nup 153 and 214 whereas, the translocation of activated STATs occurs via interaction of nuclear localization signals (sequence motifs present on surface of STAT molecules) with importins. The phosphorylated STAT3 dimer can then bind to the STAT consensus sequence to trigger transcription of various downstream genes [21,22,23,24].

S727 phosphorylation in STAT3α isoform, necessary for activation, also seems to have an intrinsic regulation mechanism for limiting STAT3 activation through dephosphorylation of Y705 by the nuclear TC45 phosphatase [15]. STAT3β, though lacking the TAD, not only modulates transcription of various genes but can also regulate the activity of α isoform [25]. Apart from this, three other mechanisms negatively regulate STAT3 signaling: (1) Suppressor Of Cytokine Signaling (SOCS) family by either direct inhibition of JAK activity (SOCS1 and SOCS3) or by competing with STAT-SH2 domains for phosphorylation by upstream kinases [26]; (2) Protein Inhibitor of Activated STATs (PIAS) by preventing STAT3 from binding to DNA; and (3) Protein tyrosine phosphatases (PTP) such as Src homology region 2 domain- containing phosphatase 1 and 2 (SHP1, SHP2), Receptor-type tyrosine-protein phosphatase T and delta (PTPRT, PTPRD) by dephosphorylating STAT3 and thereby deactivating it [27,28,29].

## 3. Role of STAT3 in Tumorigenesis: Solid and Hematological Tumors

STAT3 was first exhibited to play a role in cancer growth by Yu et al. in 1995, whose study suggested that the STAT3 activation can lead to regulation of oncogene expression [30]. Since then aberrant STAT3 expression has documented in squamous cell carcinoma of the head and neck and other solid tumors; and more recently, in haematologic malignancies [31,32,33]. Constitutively active STAT3 has been established to trigger deregulated gene transcription where the gene products consequently promote tumor progression. Altogether, STAT3 and its downstream target genes not only promote proliferation (*Cyclin D1*, *c-Myc*), survival (*Bcl-2*, *survivin, Bcl-xL*), angiogenesis (*VEGF*, *HIF1α*), and metastasis (*MMPs*), but also inhibit anti-tumor immune responses [3,34]. Conversely, interruption of STAT3 activation in the cultured cells has been found to reverse these effects [3]. Moreover, abrogation of STAT3 signalling has also exhibited tumor growth impairment and induction of apoptosis in preclinical models [35,36]. Collectively, studies have shown that close to 70% of all solid and hematological tumors exhibit aberrant STAT3 expression and/or activation [7,31], thus indicating its pivotal role in tumorigenesis. 

### 3.1. Role of STAT3 in Leukaemia

STAT3 has an important part in promoting leukemogenesis in hematopoietic stem cells. Its overexpression has been seen in malignant hematopoietic stem cells (HSCs) in Acute Myeloid Leukemia (AML) and Myelodysplastic syndrome MDS, thus insinuating that STAT3 plays a crucial role in the development of malignancies [37]. Constitutive phosphorylation and activation of STATs have been found in several leukemias [38,39] including AML [40], acute promyelocytic leukemia [41], acute lymphoblastic leukemia (ALL) [42], chronic lymphocytic leukemia (CLL) [43], and chronic myelogenous leukemia (CML) [40]. 

STAT3 has been shown to play a critical function in promoting tumor growth, increasing survival of cancerous cells, promoting resistance to chemotherapy, and linked with an increased likelihood of AML relapse in patients [32,44]. The SH2 domain of STAT3 has been found to be mutated in patients with large granular lymphocytic leukemia as well as other types of leukemias, thus indicating that anomalous STAT3 signaling may be underlying the pathogenesis of these diseases [45,46]. In 2014, Shastri et al. revealed that patients with increased STAT3 expression in HSCs were associated with a median survival of 2.6 years, while patients with lower STAT3 expression were observed to have a median survival of 5.8 years [47]. 

### 3.2. Role of STAT3 in Lymphoma

Lymphoma is defined as the solid tumor of lymphocytes and can be broadly classified into two categories: Hodgkin’s Lymphoma (HL) and Non-Hodgkin’s Lymphomas (NHL) [48]. Gene expression profiling and genomic sequence studies clearly implicate inappropriate activation of the JAK/STAT signaling in the pathogenesis of a number of lymphoma sub-types, including diffuse large B-cell lymphoma, HL, and primary mediastinal B-cell lymphoma [49].

Hodgkin’s lymphoma (HL) manifests constitutive activation of the JAK-STAT signaling pathway in the neoplastic cells which, in classical HL, are called Hodgkin’s Reed–Sternberg (HRS) cells [36,50]. Constitutive phosphorylation of STAT1 and STAT3 has been found in several HL cell lines, as well as primary HRS cells [51,52], leading to inappropriate expression of downstream genes such as *Bcl-2* and *Bcl-xL*; that have been implicated in cell survival and decreased susceptibility to apoptosis [53]. Also, studies have shown that abrogation of the JAK/STAT pathway can induce cell-arrest and apoptosis in vitro [53], inhibit tumor growth, achieve tumor elimination with significantly improved survival in murine models of human HL [54], and can significantly improve progression-free survival time specifically in patients with relapsed or treatment refractory HL [55].

NHL make up around 90% of all malignant lymphomas [56] and are generally classified according to their origin, that is either B-cell NHL or T/NK-cell NHL [57]. In a recent study, Zhang et al. have indicated that JNK and JAK/STAT pathways could stimulate cell proliferation and xenograft growth in various NHL cell lines and patient derived samples by dysregulating the expression of Insulin Enhancer Binding Protein 1 (ISL-1). The study also demonstrated that p-STAT3/p-c-Jun/ISL-1 formed a transcriptional complex that could bind directly to the ISL-1 promoter, causing its aberrant expression [57]. Involvement of the STAT3 oncogene has also been reported in diffuse large B-cell lymphoma (DLBCL) [58] and inhibition of STAT3 by targeted oligonucleotides have been shown to exert a therapeutic effect against human DLBCL xenotransplants in nude mice and to inhibit their growth by a direct cytotoxic/cytostatic effect [59]. In T-cell lymphomas such as primary cutaneous T-cell lymphomas (CTCL) and anaplastic large T-cell lymphoma (ALCL), both STAT3 and STAT5 are deregulated, and the targeting these two proteins can inhibit tumor pathogenesis in vitro [60]. 

### 3.3. Role of STAT3 in Multiple Myeloma (MM)

Multiple myeloma (MM) is the second most common hematological malignancy, marked by an accumulation of abnormal clonal plasma cells in the bone marrow [61]. Several major signalling pathways have been implicated in the pathogenesis of MM including the JAK-STAT3, PI3K/Akt/mTOR, and NF-κB pathways [62,63], wherein STAT3 might be constitutively active or may be activated by the interleukin-6(IL-6)-JAK-STAT3 axis in MM [64]. STAT3 activation has been reported to contribute to MM progression both directly by upregulating survival and anti-apoptotic target genes [65], as well as indirectly by activating myeloid derived suppressor-cells (MDSCs) in the bone marrow (BM) microenvironment, which causes T-cell suppression (immunosuppression) and facilitates tumor progression [66]. Also, Jung et al. showed that phosphotyrosine-STAT3 expression is correlated with poor prognosis and survival in MM patients [67]. Recent studies have implied that inhibition of STAT3 signalling can inhibit tumor growth, re-sensitize them to therapy, and induce apoptosis under both in vitro [68,69] and in vivo [70] conditions.

## 4. STAT3 as an Anti-Cancer Target and Selected Inhibition Strategies in Hematological Malignancies

As discussed above, most hematological malignancies manifest elevated levels of constitutively activated STAT3 and enhanced downstream transcriptional profiles in accordance with STAT3 regulated genes [13,14,71,72]. Tumor cell lines with constitutively activated STAT3 are dependent on its sustained activation, a phenomenon that has been termed “oncogene addiction” [14]. Although STAT3 activity has been shown to be imperative for embryonic development [17], it might be not be indispensable for normal or non-transformed cells and tissues [13,14], thereby making it a potential target for novel therapeutic development. Also, on a molecular structure level, STAT3, unlike other transcription factors, has unique and different domains (as described above). These sites present potential targets to modulate or inhibit STAT3 activation using multiple strategies [73]. Also, the findings that a variety of mechanisms may be involved in inappropriate activation of this pathway (Table 1), suggest its importance in hematological malignancies and, thus, make it an attractive target for therapeutic intervention [21,74,75,76,77,78,79,80,81,82,83,84].

### 4.1. Strategies for STAT3 Inhibition

**Figure 3:**
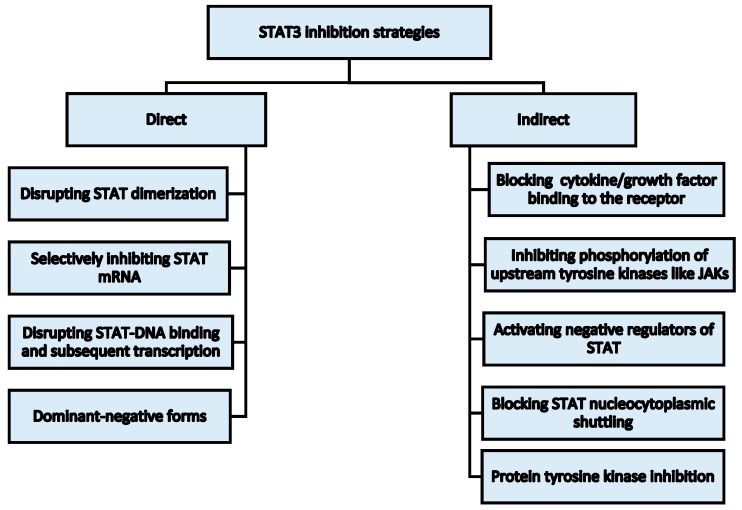
Various potential strategies for STAT3 inhibition.

Broadly, two strategies can be employed for STAT3: (1) Direct inhibition and (2) Indirect inhibition (Figure 3). Various targeted therapeutics have been developed based on one or more of these strategies. Few selected pharmacological agents targeted at inhibiting the STAT3 signaling cascade for treating various hematological malignancies are discussed in the following sections. The inhibitors may be natural or synthetic compounds, including small molecules and nucleotides [85]. Some of the inhibitors have also entered clinical trials for treatment or management of hematological malignancies and are reviewed briefly in Table 2.

#### 4.1.1. Selected Natural Compounds as Examples of STAT3 Signaling Inhibitors

##### Bavachin

Bavachin is a plant estrogen-like compound purified from plants such as *Psoralea corylifolia*. Besides its estrogen-like activities, it has also demonstrated anti-tumor and anti-bacterial effects [86]. It has been shown that bavachin abated the viability of MM cell lines in a time- as well as concentration-dependent fashion. Additionally, it also interrupted NF-κB and STAT3 phosphorylation. Moreover, it also upregulated the expression of p53 as well as NOXA and downregulated that of XIAP, survivin, Bcl-xL, and Bcl-2 proteins. Bavachin further impelled apoptosis by caspase-3/9 activation and decreased the mitochondrial membrane potential. The compound also exhibited tumor-specific activity as the viability of normal cells was not affected [69].

##### Butein

Butein is a tetrahydroxychalcone derived from plants related to cashews. It has been demonstrated to have a STAT3 inhibitory effect in various cell types including multiple myeloma (MM) cells. Deterioration of both constitutive and inducible STAT3 activation in MM cells by butein is shown to be mediated through the interruption of the upstream kinases c-Src and JAK 1&2 [87,88]. Butein was also able to act synergistically with thalidomide and bortezomib, the two first line chemotherapeutic agents for the treatment of MM, in inducing apoptosis in tumor cells. Many previous studies have shown that butein is efficacious at a low drug concentration in murine xenograft models [89].

##### Celastrol

Derived from the plant *Tripterygium wilfordii*, celastrol has been used in traditional Chinese medicine since time immemorial. In the modern context, it has been demonstrated to exert anti-cancer effects in various cancer models such as MM, AML, and hepatocellular carcinoma [68,90,91]. Celastrol suppressed constitutive as well as inducible STAT3 activation in MM cells, and the effect has been attributed to inhibition of c-Src and JAK2 activation. This abrogation induced apoptosis, lead to an increase in the aggregation of cells in the sub-G1 phase, and activation of caspase-3, thereby inducing cell death [68]. It has been also demonstrated that celastrol abates the growth of t(8:21) AML cells by activating both extrinsic and intrinsic apoptotic pathways. Additionally, celastrol decreases the expression of AML1-ETO and C-KIT at both transcriptional and translational levels, leading to suppression of AKT, STAT3, and Erk1/2 activation and their downstream signaling [90]. However, future studies in appropriate preclinical models are required to further elucidate the anti-cancer effects of celastrol in vivo and examine its clinical toxicology/efficacy.

##### Cucurbitacins

Cucurbitacins are an imperative part of herbs used in many traditional Chinese medicines [92] and have been shown to possess multiple and varied biological and medicinal properties ranging from anti-inflammatory to anti-microbial [93]. The inhibitory effect of cucurbitacin B on STAT3 was examined in the leukemia cell line K562. Following treatment with cucurbitacin B in nanomolar doses, growth inhibition and apoptosis in these leukemic cells followed a G_2_/M cell cycle arrest. The cells exhibited inhibition of STAT3 phosphorylation in a dose-dependent manner. However, the exact mechanism of STAT3 abrogation in these cell lines remains unclear, although diverse molecules have been found to mediate STAT3 suppressive effects of cucurbitacin B in other solid tumors [92,94].

##### Guggulsterone (GS)

The anti-leukemic effects of GS was initially reported by Samudio et al. [95] when they investigated the anti-cancer effects of three pregnadienedione steroidal molecules (i.e., cis-GS, trans-GS, and 16-dehydroprogesterone) in cultured leukemia cells and primary leukemic blast cells [95]. They reported that the treatment of HL-60 leukemia and U937 lymphoma cell lines with these compounds resulted in caspase-independent apoptosis and a reduction in cell proliferation. All the compounds were shown to induce generation reactive oxygen species (ROS), which could be one possible pathway for apoptosis induction. Furthermore, the compounds caused the degradation of constitutive extracellular signal-regulated kinase mediated phosphorylation of STAT3 [95]. The study by Shishodia et al. [25] observed that the treatment of leukemia, myeloma, and melanoma cell lines with GS decreased cell proliferation and caused a reduction in levels of cyclin D1 and cdc2. They also found an increased expression of p21 and p27 proteins along with induction of apoptosis by activation of PARP cleavage [25]. Another study further demonstrated that GS inhibited the constitutive activation of STAT3 in U266 myeloma cells in a dose- and time-dependent manner. In the same study, Ahn et al. also showed that GS decreased STAT3 DNA binding activity and IL-6-induced STAT3 phosphorylation. It was further determined using sodium pervanadate that GS could inhibit STAT3 activation by inducing the increased expression of a protein tyrosine phosphatase SHP1. It was also shown that GS treatment could significantly suppress the expression of proteins such as cyclin D1, Bcl-2, Bcl-xL, Mcl-1, and VEGF in a time-dependent manner [96].

##### Honokiol (HNK)

Honokiol (HNK), a biphenolic small molecular weight natural product extracted from the bark and leaves of *Magnolia officinalis*, is widely acknowledged as an antimicrobial and anti-inflammatory in traditional Chinese medicinal practice [97]. In multiple studies the compound has been shown to be effective against various hematological malignancies such as MM, T-cell leukemia, B- cell CLL, and AML [98,99,100,101]. HNK has been found to decrease the levels of phosphorylated STAT3 but does not affect total STAT3 expression. It has been shown to exhibit this effect through SHP1 expression upregulation. Additionally, HNK has been reported to inhibit transcriptional activity of STAT3, reduce its nuclear translocation, and decrease the expression of STAT3 regulated genes. The knockdown of SHP1 by small hairpin RNA (shRNA) or its inhibition with pervanadate, abolished the HNK-induced STAT3 inhibition, indicating that HNK may act via SHP1 regulation. Furthermore, HNK upregulated the expression of transcription factor PU 1, which reportedly can activate the expression of SHP1. PU 1 knockdown caused a reversal of HNK-induced upregulation of SHP1 and inactivation of STAT3 signaling. A similar increased expression of PU 1 and SHP1 in hematopoietic progenitors isolated from patients with AML was observed following HNK treatment ex vivo [99]. HNK was also able to induce the degradation of AML1-ETO in leukemic cell lines and primary AML blast cells having t(8;21) translocation. Interestingly, HNK also augmented the expression of UbcH8, an E2-conjugase for the degradation of AML1-ETO, by causing acetylation of histones in its promoter region [102]. 

##### Withaferin A

Withaferin A (WFA) is isolated from the plant *Withania somnifera* and shown to have antiproliferative properties in several cancer types. It has also been demonstrated to have other medicinal effects such as anti-inflammatory, anti-oxidant, and cardiovascular protection [103]. In the context of hematological malignancies, the compound has been shown to be a direct inhibitor of STAT3 signaling as it binds to STAT3 near the Tyr 705 phosphorylation site, thereby preventing its dimerization and hence its subsequent signaling cascade [104]. The findings warrant further investigation and development of the drug for clinical trials.

#### 4.1.2. Synthetic Inhibitors

Natural compounds have demonstrated promising effects in laboratory settings; however, it would take considerable time for them to reach clinics for widespread patient use. Although these compounds have promising low toxicity profiles, they face challenges such as specificity of targets, potency and efficacy, and their mechanisms of action being undefined or understudied. However, there are available a host of chemically synthesized molecules that exhibit promising STAT3 inhibiting activity and, in many cases, can abrogate tumor growth. Here, we describe some of these synthetic molecules with relevance to their use in hematological malignancies and their ability to act via direct or indirect STAT3 inhibition.

##### Direct Inhibitors of STAT3 Activation

Direct inhibitors, as the name suggests, interact directly with the STAT3 domains to antagonize STAT3 activation. These small molecules mainly interact with either the amino-terminal domain, SH2 domain, or DNA-binding carboxy terminal domain of STAT3 molecules. The compounds showing promising activity in targeting STAT3 in hematological malignancies include PY*LKTK, PY*L, AY*L [105], and ISS 610 [106], which are essentially small molecule peptidomimetics that interact with the SH2 domain, thereby preventing STAT dimerization and subsequent nuclear translocation and transcription. Another class of direct inhibitors being used are the G-quartets (guanosine-rich oligodeoxynucleotides) and decoy oligonucleotides. These guanosine-rich oligodeoxynucleotides were originally identified at the 3′ end of telomeres, where they were found to inhibit telomerase activity, and form intra- and inter-molecular four-stranded structures. These structures engage with the DNA binding domain of STAT3 and block protein–DNA binding. For example, molecule T40214 has been found to be a promising candidate in ALL [107]. Decoy nucleotides also work in a similar fashion as they carry the consensus STAT-binding sequences, thereby preventing the protein from binding to the STAT3 response element within the promoter [59]. Oligonucleotide mediated inhibition of STAT3 has also been achieved by using antisense oligonucleotides against STAT3 mRNA. A prime example of this class of therapeutics is AZD 9150 which exhibited antitumor activity in treatment refractory lymphoma patients in phase I study [108]. Another class of molecules using direct interaction with STAT3 proteins are chemotherapeutics. The novel platinum (IV) compound IS3 295 has been shown to irreversibly bind to the DNA binding domain of STAT3 in both its active and inactive form, and hence, preventing STAT3 from binding to DNA. The compound also exhibited suppression of STAT3 induced genes *Bcl-xL* and *cyclin D1* [109]. More direct STAT3 inhibitors are in development, such as the dual-function molecule CpG-STAT3dODN, which consists of a TLR9 (Toll like receptor) agonist (CpG7909) and a STAT3 inhibitor, high-affinity decoy oligodeoxynucleotide for B-cell lymphoma immunotherapy [59].

##### Indirect Inhibitors

STAT3 is a multistep signaling cascade involving a multitude of components. This provides an opportunity for the development of compounds that can interact with other constituents of the pathway and thus abrogate STAT3 activity in a defined manner. One of the foremost approaches to indirect STAT3 inhibition is receptor antagonism. As STATs are downstream to several cytokine and growth receptors, especially in MM and leukemias, monoclonal antibodies blocking these receptors represent a promising choice. As a proof of concept, the chimeric antibody rituximab has shown inhibition of constitutive STAT3 activity by blocking the CD-20 receptor in Non-Hodgkin’s Lymphoma. The blockage was demonstrated to downregulate IL-10, leading to STAT3 inhibition [110].

In MM, JAKs can be activated by IL-6 present in the bone marrow micro-environment. This leads to recruitment of two gp130 signal–transducing subunits to the receptor complex; consequently, gp130-associated JAKs (i.e., JAK1, JAK2, and Tyk2) become activated to provide docking sites for STAT3 [111]. Therefore, specific tyrosine kinase inhibitors have caught focal attention as agents for disruption of STAT activation. Selective and specific cessation of JAK2 activity has been demonstrated using AG490 and its congeners LS-104 [112], CEP701, and ICNB18424, and has been shown to inhibit the growth of leukemia cells both in vitro and in vivo [113,114]. LS-104 is currently in phase II clinical trials for the treatment of ALL and has shown promising STAT3 inhibition in vitro in FLT3 positive AML [113,115]. NCB1824, a small-molecule inhibitor of both JAK1 and JAK2, suppresses levels of phosphorylated STAT3 in subjects with wild-type JAK2 or with the gain-of-function V^617^F JAK2 mutation, as revealed in a phase 1–2 clinical trial of myelofibrosis patients. Another synthetic compound INCB20 potently disrupts the activation of all members of the JAK family, and thus blocks the IL-6 mediated progression of MM. It also blocks SH-2 phosphorylation, thus abrogating cytokine mediated dexamethasone resistance [116].

Other than JAK activation, elevated levels and/or kinase activity of Src and other growth factor receptors have been indicated in several cancers, causing activation of STAT3. These also make important potential targets to check STAT3 activity. One such molecule is Dasatinib, which can inhibit SFK and BCR/ABL activation and has been approved for use as second line treatment in patients with chronic myelogenous leukemia and also for Philadelphia chromosome–positive ALL [38]. Although they are a druggable components of the STAT3 cascade, the relatively modest response rates of tumors to therapeutic agents that target the EGFR, JAKs, and SFKs illustrate that the inhibition of single pathways may be insufficient to completely block the aberrant activation of STAT3 [85].

Targeting STAT nuclear translocation is yet another potential approach to develop efficacious anti-cancer drugs. The activity of phosphorylated STAT3 as a DNA-binding transcription factor depends upon the trafficking of homodimers from cytosol and into the nucleus [117]. One such compound, Selinexor, has exhibited potential clinical activity in AML and MM [118]. More such inhibitors are in the pipeline to be further examined and understood but they present a promising class of STAT3 inhibitors.

STAT3 proteins exhibit negative regulation by 3 major protein families: (1) Phosphotyrosine phosphatase (PTP) which regulates its activity by dephosphorylating upstream kinases [119]; (2) PIAS, and (3) SOCS, which are negative regulators of STAT3 signaling [120,121]. Specific demethylation agents seem to be an interesting class of STAT3 regulators, since hypermethylation can silence these protein regulators in AML.

## 5. Conclusions and Future Perspectives

Several hallmarks of cancer, such as uncontrolled cellular proliferation, invasion/metastasis, and resistance to apoptosis, are associated with dysregulated STAT signaling. In the context of hematological malignancies, aberrant STAT3 activation becomes an even more important factor in tumor pathogenesis due to the constant presence of cytokines and growth factors in the bone marrow microenvironment. Although not the only transcription factor involved in tumorigenesis, its inhibition has been shown to prevent malignant transformation in leukemias and lymphomas. Elucidation of multifaceted STAT3 signaling cascade using multi-omics approaches can lead to deeper understanding of its pathological role in the development of malignancies and their progression.

In conclusion, it can be reconciled that STAT3 has been validated as a druggable target and important pathway in cancer therapeutics. Our lab and other pharma-oncology groups are working with various classes of pharmacological blockers of STAT3 signaling, and important advancements have been made in recent years to develop clinically relevant drug candidates for STAT3 inhibition. Although it remains a challenge to develop a truly potent and specific STAT3 inhibitor, the current compounds can serve as great leads to develop better and efficacious anticancer therapies.

## Figures and Tables

**Table 1:** Selected molecular mechanisms of aberrant activation of STAT3 in hematological malignancies.

Hematological Malignancy	Subtype(s)	Activation Mechanism(s)	References
Acute Leukaemias	ALL	BCR/abl fusion protein	[76]
		TEL-JAK2 fusion protein	[77]
	AML	Aberrant exogenous cytokine signaling	[75]
		Constitutive Protein Tyrosine Kinases (PTK) activation without exposure to exogenous cytokines	[75]
		Activating STAT3 mutations	[21]
		Hypermethylation, hence, silencing of negative regulators of STAT3 (PIAS3, SOCS3 and PTP)	[21]
Chronic Leukemias	CLL	Casein Kinase2(CK2)-B cell linker (BLNK)-CD5 complex causes constitutive phosphorylation	[79]
	CML	JAK activation by bone marrow microenvironment	[78]
Lymphomas	HL	Activating mutations in JAK1 and STAT3	[80]
		Autocrine secretion of IL13 by HRS cells	[82]
	NHL	Autocrine and paracrine secretion of IL6 and IL13	[81,83]
Multiple Myeloma	-	Autocrine and paracrine secretion of IL6 and subsequent activation of JAK1	[62]
		Overexpression and hyperactivation of CK2	[84]

**Table 2:** Selective STAT3 inhibitors in clinical trials for hematological malignancies.

Inhibitor	Indication	Status	References
Dasatinib (Tyrosine Kinase Inhibitor)	ALL, CML	FDA approved, specifically for Ph+ cases	[122]
Imatinib (Bcr-Abl Tyrosine Kinase Inhibitor)	CML, ALL	FDA approved, specifically for Ph+ cases	[123]
Ruxolitinib (JAK 1&2 inhibitor)	Myeloproliferative neoplasms	FDA approved for Myelofibrosis and as second line treatment for Polycythemia vera	[124]
	AML	Phase I/II trial terminated due to lack of efficacy	[125]
Pyrimethamine (Direct STAT3 SH2 domain inhibitor)	CLL	Phase I/II clinical trials	[126]
AZD 9150 (STAT3 Antisense oligonucleotide)	Lymphoma	Phase I dose escalation study completed	[108]
OPB 31121 (STAT3 SH2 domain inhibitor)	MM, NHL Leukemia	Trial terminated due to high toxicity and poor pharmacokinetics	[126]
OPB 51602 (STAT3 SH2 domain inhibitor)	MM, AML NHL, CML	Trial terminated due to inefficacy in hematological malignancies	[126]
